# *catena*-Poly[[[bis­(μ-4-hydroxy­benzoato)bis­[(4-hydroxy­benzoato)manganese(II)]]-di-μ-4,4′-bipyridine] 4,4′-bipyridine disolvate tetra­hydrate]

**DOI:** 10.1107/S1600536810007245

**Published:** 2010-03-03

**Authors:** Qi-Hua Zhao, Lei Zhao, Kun-Miao Wang, Hong Zhou

**Affiliations:** a School of Chemical Science and Technology, Key Laboratory of Medicinal Chemistry for Natural Resource, Ministry of Education, Yunnan University, Kunming 650091, People’s Republic of China; b College of Basic Science and Information Engineering, Yunnan Agricultural University, Kunming 650201, People’s Republic of China

## Abstract

In the polymeric title complex, {[Mn(O_2_CC_6_H_4_-*p*-OH)_2_(C_10_H_8_N_2_)]·C_10_H_8_N_2_·2H_2_O}_*n*_, the Mn^II^ atom is coordinated in a distorted octa­hedral geometry defined by four O atoms from three different carboxyl­ate ligands and two *trans*-N atoms from two 4,4′-bipyridine ligands. In the crystal structure, an extensive range of O—H⋯O and O—H⋯N hydrogen bonds link the complex and all non-coordinated mol­ecules into a three-dimensional network.

## Related literature

For background to the use of aromatic carboxyl­ates and 4,4′-bipyridine in the design of supra­molecular structures containing metal-organic mol­ecules, see: Leonard *et al.* (1998 ▶); Lucia *et al.* (1997 ▶); Corey *et al.* (2001 ▶). 
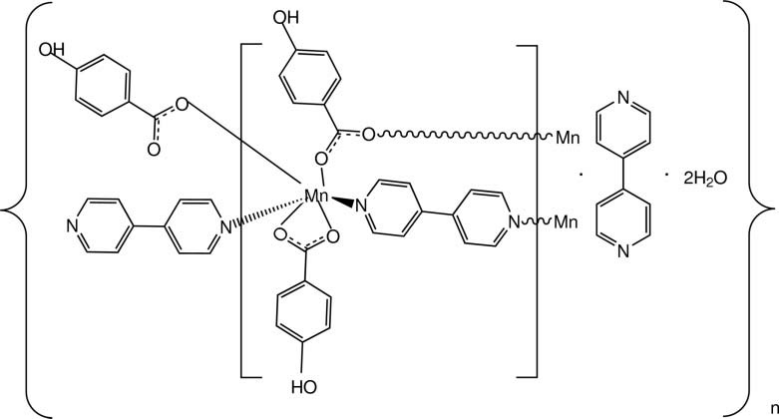


## Experimental

### 

#### Crystal data

[Mn(C_7_H_5_O_3_)_2_(C_10_H_8_N_2_)]·C_10_H_8_N_2_·2H_2_O
*M* *_r_* = 677.56
Monoclinic, 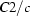

*a* = 18.1031 (17) Å
*b* = 11.6448 (11) Å
*c* = 31.771 (3) Å
β = 104.957 (1)°
*V* = 6470.6 (11) Å^3^
*Z* = 8
Mo *K*α radiation
μ = 0.47 mm^−1^
*T* = 293 K
0.20 × 0.13 × 0.10 mm

#### Data collection

Bruker APEXII 1K CCD area-detector diffractometer
Absorption correction: multi-scan (*SADABS*; Sheldrick, 2004 ▶) *T*_min_ = 0.931, *T*_max_ = 0.955
20618 measured reflections
7526 independent reflections
4072 reflections with *I* > 2σ(*I*)
*R*_int_ = 0.055

#### Refinement

*R*[*F* ^2^ > 2σ(*F* ^2^)] = 0.051
*wR*(*F* ^2^) = 0.130
*S* = 1.01
7526 reflections
428 parameters
7 restraints
H-atom parameters constrained
Δρ_max_ = 0.48 e Å^−3^
Δρ_min_ = −0.34 e Å^−3^

### 

Data collection: *APEX2* (Bruker, 2004 ▶); cell refinement: *SAINT* (Bruker, 2004 ▶); data reduction: *SAINT*; program(s) used to solve structure: *SHELXS97* (Sheldrick, 2008 ▶); program(s) used to refine structure: *SHELXL97* (Sheldrick, 2008 ▶); molecular graphics: *SHELXTL* (Sheldrick, 2008 ▶); software used to prepare material for publication: *SHELXTL*.

## Supplementary Material

Crystal structure: contains datablocks I, global. DOI: 10.1107/S1600536810007245/tk2614sup1.cif

Structure factors: contains datablocks I. DOI: 10.1107/S1600536810007245/tk2614Isup2.hkl

Additional supplementary materials:  crystallographic information; 3D view; checkCIF report

## Figures and Tables

**Table 1:** Hydrogen-bond geometry (Å, °)

*D*—H⋯*A*	*D*—H	H⋯*A*	*D*⋯*A*	*D*—H⋯*A*
O1*W*—H1*WA*⋯O2*W*	0.94	2.00	2.781 (4)	140
O1*W*—H1*WB*⋯O2*W*^i^	0.97	2.15	2.992 (4)	145
O2*W*—H2*WB*⋯N4	0.89	2.34	2.934 (4)	124
O2*W*—H2*WA*⋯O2	0.98	1.82	2.793 (3)	172
O3—H3*B*⋯N3^ii^	0.82	1.88	2.694 (4)	169
O6—H6*B*⋯O1*W*^iii^	0.82	1.90	2.701 (4)	167

Symmetry codes: (i) 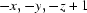; (ii) 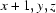; (iii) 
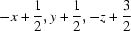
.

**Fig. 1.**
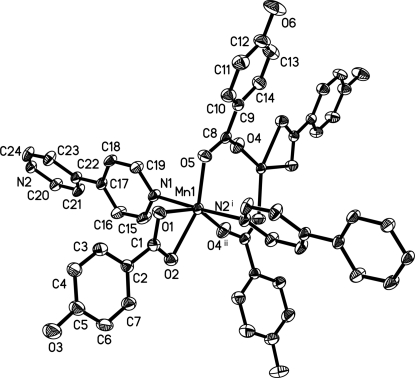
The immediate environment for the Mn atom in the title complex with the atom-numbering shown for the asymmetric unit. Non-coordinated molecules and hydrogen atoms are omitted for clarity. Displacement ellipsoids were drawn at the 30% probability level.

**Fig. 2.**
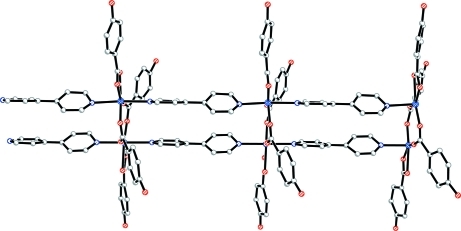
The 1D double chain in the title complex. Non-coordinated molecules and hydrogen atoms are omitted for clarity.

## supplementary crystallographic information

### Comment

Much progress has been achieved in the design of supramolecular structures containing metal-organic molecules during recent years. Multifunctional ligands can link metal ions into one-, two- or three-dimensional structures, and in this context, aromatic carboxylates and 4,4'-bipyridine have been used successfully to synthesize such materials (Leonard *et al.*, 1998; Lucia *et al.*, 1997; Corey *et al.*, 2001).

As shown in Fig. 1, the Mn^II^ coordination environment can be considered as a distorted octahedral geometry, being coordinated by four O atoms in the equatorial plane (O1, O2, O5, O4^ii^; ii = -x, y, -z+3/2), derived from three different carboxylate groups, and two trans-N atoms (N1, N2^i^; i = x, y+1, z). The Mn—O chelating bond distances (2.271 (2) Å and 2.281 (2) Å) are longer than Mn—O bridging bonds (2.087 (2) Å and 2.1102 (19) Å). Pairs of Mn ions are linked via two carboxyl groups to generate an eight-membered Mn_2_(COO)_2_ ring with a Mn···Mn distance of 4.173 (2) Å. These Mn_2_(COO)_2_ units are further connected by two 4,4'-bipyridine ligands to form a double-chain structure (Fig. 2) with a Mn—Mn distance 8.355 (4) Å. There are six kinds of H-bonds occurring between the chains, the solvent water and 4,4'-bipyridine molecules, which join the components of the structure into a 3-D network.

### Experimental

Mn(O_2_CCH_3_)_2_ (27.67 mg, 0.1 mmol) was dissolved in H_2_O (5 ml), and 4-hydroxybenzoic acid (27.67 mg, 0.2 mmol) was dissolved in methanol (5 ml) at room temperature. The mixture was stirred for 1 h, a methanol solution (5 ml) of 4,4'-bipyridine (31.28 mg, 0.2 mmol) was added, and stirring continued for 30 mins. Yellow single crystals of the title complex were obtained by slow evaporation at room temperature for two weeks.

### Refinement

H atoms bonded to O1W and O2W atoms were located in a difference map and fixed at those positions (see Table 1 for bond distances) but their U_iso_ values were refined. The remaining H atoms were calculated geometrically and were allowed to ride on the parent atoms with distance restraints of O—H = 0.82 Å and C—H = 0.93 Å, and with *U*_iso_(H) = 1.5U_eq_(O) and 1.2U_eq_(C).

### Crystal data

**Table:**

[Mn(C_7_H_5_O_3_)_2_(C_10_H_8_N_2_)]·C_10_H_8_N_2_·2H_2_O	*F*(000) = 2808
*M**_r_* = 677.56	*D*_x_ = 1.391 Mg m^−^^3^
Monoclinic, *C*2/*c*	Mo *K*α radiation, λ = 0.71073 Å
Hall symbol: -C 2yc	Cell parameters from 2470 reflections
*a* = 18.1031 (17) Å	θ = 2.3–20.7°
*b* = 11.6448 (11) Å	µ = 0.47 mm^−^^1^
*c* = 31.771 (3) Å	*T* = 293 K
β = 104.957 (1)°	Irregular, yellow
*V* = 6470.6 (11) Å^3^	0.20 × 0.13 × 0.10 mm
*Z* = 8	

### Data collection

**Table:**

Bruker APEXII 1K CCD area-detector diffractometer	7526 independent reflections
Radiation source: fine-focus sealed tube	4072 reflections with *I* > 2σ(*I*)
graphite	*R*_int_ = 0.055
φ and ω scans	θ_max_ = 28.2°, θ_min_ = 2.1°
Absorption correction: multi-scan (*SADABS*; Sheldrick, 2004)	*h* = −23→17
*T*_min_ = 0.931, *T*_max_ = 0.955	*k* = −15→14
20618 measured reflections	*l* = −27→41

### Refinement

**Table:**

Refinement on *F*^2^	Primary atom site location: structure-invariant direct methods
Least-squares matrix: full	Secondary atom site location: difference Fourier map
*R*[*F*^2^ > 2σ(*F*^2^)] = 0.051	Hydrogen site location: inferred from neighbouring sites
*wR*(*F*^2^) = 0.130	H-atom parameters constrained
*S* = 1.01	*w* = 1/[σ^2^(*F*_o_^2^) + (0.0516*P*)^2^] where *P* = (*F*_o_^2^ + 2*F*_c_^2^)/3
7526 reflections	(Δ/σ)_max_ = 0.002
428 parameters	Δρ_max_ = 0.48 e Å^−^^3^
7 restraints	Δρ_min_ = −0.34 e Å^−^^3^

### Special details

**Table:**

Geometry. All esds (except the esd in the dihedral angle between two l.s. planes) are estimated using the full covariance matrix. The cell esds are taken into account individually in the estimation of esds in distances, angles and torsion angles; correlations between esds in cell parameters are only used when they are defined by crystal symmetry. An approximate (isotropic) treatment of cell esds is used for estimating esds involving l.s. planes.
Refinement. Refinement of *F*^2^ against ALL reflections. The weighted *R*-factor wR and goodness of fit *S* are based on *F*^2^, conventional *R*-factors *R* are based on *F*, with *F* set to zero for negative *F*^2^. The threshold expression of *F*^2^ > σ(*F*^2^) is used only for calculating *R*-factors(gt) etc. and is not relevant to the choice of reflections for refinement. *R*-factors based on *F*^2^ are statistically about twice as large as those based on *F*, and *R*- factors based on ALL data will be even larger.

### Fractional atomic coordinates and isotropic or equivalent isotropic displacement parameters (Å^2^)

**Table:**

	*x*	*y*	*z*	*U*_iso_*/*U*_eq_	
Mn1	0.08008 (2)	0.15824 (3)	0.713098 (13)	0.03256 (13)	
O1	0.19470 (11)	0.15352 (16)	0.69639 (6)	0.0465 (5)	
O1W	0.06897 (16)	−0.1109 (3)	0.51429 (10)	0.1000 (9)	
H1WA	0.0579	−0.0415	0.5263	0.30 (4)*	
H1WB	0.0341	−0.1120	0.4856	0.16 (2)*	
O2	0.09014 (11)	0.15001 (16)	0.64305 (6)	0.0458 (5)	
O2W	0.01136 (14)	0.0156 (3)	0.57296 (8)	0.0980 (10)	
H2WB	−0.0031	−0.0486	0.5838	0.23 (4)*	
H2WA	0.0431	0.0574	0.5981	0.144 (19)*	
O3	0.33963 (13)	0.1358 (2)	0.53460 (7)	0.0775 (8)	
H3B	0.3834	0.1140	0.5459	0.116*	
O4	0.03921 (11)	0.15809 (17)	0.80555 (7)	0.0573 (6)	
O5	0.14062 (11)	0.16769 (16)	0.77935 (6)	0.0483 (5)	
O6	0.27872 (16)	0.3557 (2)	0.97038 (8)	0.0966 (9)	
H6B	0.3241	0.3623	0.9709	0.145*	
N1	0.08390 (12)	−0.03575 (18)	0.71607 (8)	0.0399 (6)	
N2	0.08120 (13)	−0.64423 (17)	0.71374 (8)	0.0412 (6)	
N3	−0.51598 (16)	0.0563 (3)	0.56051 (11)	0.0741 (9)	
N4	−0.12265 (15)	−0.0748 (3)	0.59787 (10)	0.0636 (8)	
C1	0.16240 (16)	0.1471 (2)	0.65617 (9)	0.0384 (7)	
C2	0.20923 (16)	0.1390 (2)	0.62421 (9)	0.0386 (7)	
C3	0.28472 (17)	0.1051 (3)	0.63700 (10)	0.0484 (8)	
H3A	0.3061	0.0842	0.6658	0.058*	
C4	0.32916 (18)	0.1016 (3)	0.60746 (10)	0.0544 (9)	
H4A	0.3797	0.0771	0.6163	0.065*	
C5	0.29825 (18)	0.1346 (3)	0.56487 (11)	0.0541 (9)	
C6	0.22282 (18)	0.1687 (3)	0.55139 (10)	0.0564 (9)	
H6A	0.2020	0.1912	0.5227	0.068*	
C7	0.17852 (17)	0.1690 (3)	0.58084 (10)	0.0492 (8)	
H7A	0.1273	0.1897	0.5715	0.059*	
C8	0.10840 (16)	0.1807 (2)	0.80963 (9)	0.0352 (7)	
C9	0.15477 (15)	0.2283 (2)	0.85155 (9)	0.0359 (7)	
C10	0.22868 (17)	0.2643 (2)	0.85611 (10)	0.0499 (8)	
H10A	0.2502	0.2589	0.8326	0.060*	
C11	0.27225 (18)	0.3093 (3)	0.89598 (11)	0.0586 (9)	
H11A	0.3220	0.3347	0.8990	0.070*	
C12	0.2391 (2)	0.3146 (3)	0.93039 (11)	0.0598 (9)	
C13	0.1661 (2)	0.2796 (3)	0.92614 (11)	0.0689 (10)	
H13A	0.1446	0.2846	0.9497	0.083*	
C14	0.12380 (18)	0.2368 (3)	0.88709 (10)	0.0516 (8)	
H14A	0.0737	0.2131	0.8844	0.062*	
C15	0.03712 (17)	−0.0965 (2)	0.68436 (10)	0.0548 (9)	
H15A	0.0024	−0.0571	0.6624	0.066*	
C16	0.03788 (18)	−0.2137 (2)	0.68267 (11)	0.0567 (9)	
H16A	0.0044	−0.2518	0.6598	0.068*	
C17	0.08816 (15)	−0.2762 (2)	0.71470 (9)	0.0383 (7)	
C18	0.13833 (16)	−0.2140 (2)	0.74664 (10)	0.0459 (8)	
H18A	0.1746	−0.2514	0.7684	0.055*	
C19	0.13412 (16)	−0.0958 (2)	0.74605 (10)	0.0447 (8)	
H19A	0.1684	−0.0556	0.7679	0.054*	
C20	0.01869 (16)	−0.5805 (2)	0.69826 (10)	0.0468 (8)	
H20A	−0.0275	−0.6181	0.6868	0.056*	
C21	0.01906 (16)	−0.4630 (2)	0.69834 (10)	0.0470 (8)	
H21A	−0.0263	−0.4230	0.6875	0.056*	
C22	0.08681 (15)	−0.4033 (2)	0.71451 (9)	0.0379 (7)	
C23	0.15139 (16)	−0.4697 (2)	0.73069 (10)	0.0507 (9)	
H23A	0.1984	−0.4346	0.7422	0.061*	
C24	0.14585 (16)	−0.5868 (2)	0.72969 (11)	0.0514 (9)	
H24A	0.1902	−0.6290	0.7409	0.062*	
C25	−0.4703 (2)	0.1155 (3)	0.54225 (14)	0.0797 (12)	
H25A	−0.4910	0.1766	0.5241	0.096*	
C26	−0.3937 (2)	0.0916 (3)	0.54861 (12)	0.0715 (11)	
H26A	−0.3643	0.1349	0.5344	0.086*	
C27	−0.36040 (18)	0.0029 (3)	0.57622 (10)	0.0514 (8)	
C28	−0.40861 (19)	−0.0576 (3)	0.59569 (11)	0.0595 (9)	
H28A	−0.3896	−0.1175	0.6148	0.071*	
C29	−0.4846 (2)	−0.0285 (3)	0.58664 (12)	0.0719 (11)	
H29A	−0.5160	−0.0713	0.5997	0.086*	
C30	−0.1652 (2)	−0.0374 (3)	0.56010 (12)	0.0735 (11)	
H30A	−0.1418	−0.0279	0.5375	0.088*	
C31	−0.24157 (19)	−0.0116 (3)	0.55193 (11)	0.0686 (10)	
H31A	−0.2682	0.0145	0.5246	0.082*	
C32	−0.27841 (17)	−0.0247 (3)	0.58427 (10)	0.0498 (8)	
C33	−0.23480 (19)	−0.0645 (3)	0.62397 (11)	0.0592 (9)	
H33A	−0.2567	−0.0756	0.6471	0.071*	
C34	−0.1590 (2)	−0.0875 (3)	0.62865 (12)	0.0662 (10)	
H34A	−0.1308	−0.1141	0.6556	0.079*	

### Atomic displacement parameters (Å^2^)

**Table:**

	*U*^11^	*U*^22^	*U*^33^	*U*^12^	*U*^13^	*U*^23^
Mn1	0.0373 (2)	0.0223 (2)	0.0358 (2)	−0.00006 (18)	0.00524 (18)	−0.00108 (19)
O1	0.0462 (12)	0.0540 (12)	0.0382 (12)	−0.0019 (10)	0.0087 (10)	−0.0069 (10)
O1W	0.103 (2)	0.106 (2)	0.084 (2)	0.0188 (18)	0.0113 (19)	0.0147 (18)
O2	0.0410 (12)	0.0535 (13)	0.0409 (12)	0.0010 (10)	0.0071 (9)	−0.0006 (10)
O2W	0.0773 (19)	0.156 (3)	0.0601 (17)	−0.0445 (19)	0.0172 (15)	−0.0286 (19)
O3	0.0578 (15)	0.127 (2)	0.0515 (15)	0.0158 (14)	0.0203 (13)	0.0114 (15)
O4	0.0376 (12)	0.0491 (12)	0.0768 (16)	−0.0018 (10)	−0.0003 (11)	−0.0030 (12)
O5	0.0580 (13)	0.0488 (12)	0.0350 (11)	0.0001 (10)	0.0061 (10)	−0.0049 (10)
O6	0.118 (2)	0.110 (2)	0.0490 (16)	−0.0248 (17)	−0.0017 (15)	−0.0248 (15)
N1	0.0428 (14)	0.0241 (12)	0.0461 (15)	−0.0004 (10)	−0.0005 (11)	0.0037 (11)
N2	0.0447 (14)	0.0238 (12)	0.0501 (15)	0.0004 (11)	0.0035 (12)	−0.0037 (11)
N3	0.0562 (19)	0.092 (3)	0.078 (2)	0.0014 (18)	0.0234 (17)	0.0091 (19)
N4	0.0528 (18)	0.077 (2)	0.059 (2)	−0.0074 (15)	0.0102 (16)	−0.0033 (16)
C1	0.0439 (17)	0.0311 (15)	0.0382 (17)	−0.0037 (13)	0.0070 (14)	−0.0007 (13)
C2	0.0453 (17)	0.0333 (16)	0.0376 (17)	−0.0026 (13)	0.0114 (14)	−0.0021 (13)
C3	0.0444 (19)	0.0562 (19)	0.0400 (18)	0.0027 (15)	0.0026 (15)	−0.0011 (15)
C4	0.0407 (18)	0.079 (2)	0.043 (2)	0.0091 (17)	0.0094 (16)	−0.0028 (18)
C5	0.048 (2)	0.072 (2)	0.044 (2)	0.0008 (17)	0.0159 (17)	−0.0006 (17)
C6	0.056 (2)	0.079 (2)	0.0327 (17)	0.0038 (18)	0.0096 (16)	0.0069 (17)
C7	0.0437 (18)	0.058 (2)	0.0441 (19)	0.0032 (15)	0.0083 (15)	0.0035 (16)
C8	0.0382 (17)	0.0218 (14)	0.0408 (17)	0.0020 (11)	0.0015 (14)	0.0005 (12)
C9	0.0401 (16)	0.0296 (14)	0.0352 (16)	0.0013 (12)	0.0048 (13)	−0.0010 (12)
C10	0.0495 (19)	0.0499 (19)	0.046 (2)	−0.0045 (15)	0.0047 (15)	−0.0024 (15)
C11	0.052 (2)	0.057 (2)	0.059 (2)	−0.0117 (16)	−0.0003 (18)	−0.0025 (18)
C12	0.074 (3)	0.056 (2)	0.042 (2)	−0.0070 (18)	0.0011 (19)	−0.0054 (16)
C13	0.088 (3)	0.071 (3)	0.050 (2)	−0.006 (2)	0.023 (2)	−0.0096 (19)
C14	0.057 (2)	0.051 (2)	0.047 (2)	−0.0006 (16)	0.0128 (17)	−0.0031 (16)
C15	0.060 (2)	0.0326 (17)	0.053 (2)	−0.0013 (15)	−0.0178 (16)	0.0013 (15)
C16	0.069 (2)	0.0290 (16)	0.054 (2)	−0.0061 (15)	−0.0185 (17)	−0.0020 (15)
C17	0.0401 (16)	0.0253 (14)	0.0455 (18)	0.0015 (12)	0.0035 (14)	−0.0010 (13)
C18	0.0521 (18)	0.0268 (15)	0.0468 (19)	0.0032 (13)	−0.0089 (14)	0.0022 (14)
C19	0.0488 (18)	0.0296 (16)	0.0451 (18)	−0.0027 (13)	−0.0074 (14)	−0.0022 (14)
C20	0.0460 (18)	0.0293 (15)	0.057 (2)	−0.0057 (14)	−0.0017 (15)	−0.0003 (14)
C21	0.0410 (17)	0.0270 (15)	0.061 (2)	0.0027 (13)	−0.0077 (15)	0.0016 (14)
C22	0.0450 (17)	0.0209 (14)	0.0428 (17)	0.0008 (12)	0.0022 (14)	0.0000 (13)
C23	0.0393 (17)	0.0292 (15)	0.074 (2)	−0.0012 (13)	−0.0033 (16)	−0.0015 (15)
C24	0.0398 (18)	0.0285 (15)	0.077 (2)	0.0049 (13)	−0.0008 (16)	−0.0007 (16)
C25	0.057 (3)	0.084 (3)	0.092 (3)	0.005 (2)	0.009 (2)	0.024 (2)
C26	0.057 (2)	0.081 (3)	0.074 (3)	−0.012 (2)	0.013 (2)	0.022 (2)
C27	0.055 (2)	0.052 (2)	0.0469 (19)	−0.0095 (16)	0.0112 (16)	0.0000 (16)
C28	0.060 (2)	0.063 (2)	0.062 (2)	0.0013 (18)	0.0262 (19)	0.0100 (18)
C29	0.071 (3)	0.077 (3)	0.077 (3)	−0.004 (2)	0.035 (2)	0.006 (2)
C30	0.053 (2)	0.115 (3)	0.054 (2)	−0.016 (2)	0.0187 (19)	−0.009 (2)
C31	0.048 (2)	0.103 (3)	0.050 (2)	−0.018 (2)	0.0041 (18)	0.006 (2)
C32	0.0447 (19)	0.0517 (19)	0.051 (2)	−0.0126 (15)	0.0084 (17)	−0.0018 (16)
C33	0.062 (2)	0.065 (2)	0.050 (2)	−0.0018 (18)	0.0137 (18)	0.0075 (17)
C34	0.063 (2)	0.077 (3)	0.052 (2)	0.002 (2)	0.0014 (19)	0.0085 (19)

### Geometric parameters (Å, °)

**Table:**

Mn1—O4^i^	2.087 (2)	C10—C11	1.409 (4)
Mn1—O5	2.1102 (19)	C10—H10A	0.9300
Mn1—N1	2.261 (2)	C11—C12	1.377 (5)
Mn1—O1	2.271 (2)	C11—H11A	0.9300
Mn1—O2	2.281 (2)	C12—C13	1.357 (4)
Mn1—N2^ii^	2.300 (2)	C13—C14	1.372 (4)
Mn1—C1	2.627 (3)	C13—H13A	0.9300
O1—C1	1.263 (3)	C14—H14A	0.9300
O1W—H1WA	0.9383	C15—C16	1.366 (4)
O1W—H1WB	0.9656	C15—H15A	0.9300
O2—C1	1.267 (3)	C16—C17	1.384 (4)
O2W—H2WB	0.8897	C16—H16A	0.9300
O2W—H2WA	0.9833	C17—C18	1.380 (4)
O3—C5	1.363 (4)	C17—C22	1.480 (3)
O3—H3B	0.8200	C18—C19	1.378 (4)
O4—C8	1.253 (3)	C18—H18A	0.9300
O4—Mn1^i^	2.087 (2)	C19—H19A	0.9300
O5—C8	1.256 (3)	C20—C21	1.368 (4)
O6—C12	1.373 (4)	C20—H20A	0.9300
O6—H6B	0.8200	C21—C22	1.388 (4)
N1—C19	1.333 (3)	C21—H21A	0.9300
N1—C15	1.339 (3)	C22—C23	1.385 (4)
N2—C24	1.330 (3)	C23—C24	1.367 (4)
N2—C20	1.336 (3)	C23—H23A	0.9300
N2—Mn1^iii^	2.300 (2)	C24—H24A	0.9300
N3—C25	1.320 (4)	C25—C26	1.378 (4)
N3—C29	1.321 (4)	C25—H25A	0.9300
N4—C34	1.320 (4)	C26—C27	1.388 (4)
N4—C30	1.321 (4)	C26—H26A	0.9300
C1—C2	1.484 (4)	C27—C28	1.385 (4)
C2—C3	1.379 (4)	C27—C32	1.475 (4)
C2—C7	1.391 (4)	C28—C29	1.373 (4)
C3—C4	1.385 (4)	C28—H28A	0.9300
C3—H3A	0.9300	C29—H29A	0.9300
C4—C5	1.379 (4)	C30—C31	1.372 (4)
C4—H4A	0.9300	C30—H30A	0.9300
C5—C6	1.380 (4)	C31—C32	1.370 (4)
C6—C7	1.381 (4)	C31—H31A	0.9300
C6—H6A	0.9300	C32—C33	1.384 (4)
C7—H7A	0.9300	C33—C34	1.369 (4)
C8—C9	1.486 (4)	C33—H33A	0.9300
C9—C10	1.374 (4)	C34—H34A	0.9300
C9—C14	1.388 (4)		
			
O4^i^—Mn1—O5	121.08 (9)	C12—C11—H11A	120.9
O4^i^—Mn1—N1	91.68 (8)	C10—C11—H11A	120.9
O5—Mn1—N1	90.46 (8)	C13—C12—O6	117.3 (3)
O4^i^—Mn1—O1	150.99 (8)	C13—C12—C11	121.3 (3)
O5—Mn1—O1	87.92 (8)	O6—C12—C11	121.3 (3)
N1—Mn1—O1	88.03 (8)	C12—C13—C14	120.0 (3)
O4^i^—Mn1—O2	93.46 (8)	C12—C13—H13A	120.0
O5—Mn1—O2	145.44 (8)	C14—C13—H13A	120.0
N1—Mn1—O2	89.40 (8)	C13—C14—C9	120.9 (3)
O1—Mn1—O2	57.53 (7)	C13—C14—H14A	119.5
O4^i^—Mn1—N2^ii^	90.54 (8)	C9—C14—H14A	119.5
O5—Mn1—N2^ii^	86.44 (8)	N1—C15—C16	123.2 (3)
N1—Mn1—N2^ii^	176.82 (8)	N1—C15—H15A	118.4
O1—Mn1—N2^ii^	91.13 (8)	C16—C15—H15A	118.4
O2—Mn1—N2^ii^	92.75 (8)	C15—C16—C17	120.5 (3)
O4^i^—Mn1—C1	122.28 (9)	C15—C16—H16A	119.7
O5—Mn1—C1	116.65 (9)	C17—C16—H16A	119.7
N1—Mn1—C1	87.86 (8)	C18—C17—C16	116.6 (2)
O1—Mn1—C1	28.72 (7)	C18—C17—C22	122.4 (2)
O2—Mn1—C1	28.83 (7)	C16—C17—C22	121.1 (2)
N2^ii^—Mn1—C1	92.88 (8)	C19—C18—C17	119.4 (3)
C1—O1—Mn1	91.46 (17)	C19—C18—H18A	120.3
H1WA—O1W—H1WB	103.8	C17—C18—H18A	120.3
C1—O2—Mn1	90.90 (17)	N1—C19—C18	124.0 (3)
H2WB—O2W—H2WA	105.7	N1—C19—H19A	118.0
C5—O3—H3B	109.5	C18—C19—H19A	118.0
C8—O4—Mn1^i^	164.27 (19)	N2—C20—C21	123.4 (3)
C8—O5—Mn1	123.08 (19)	N2—C20—H20A	118.3
C12—O6—H6B	109.5	C21—C20—H20A	118.3
C19—N1—C15	116.3 (2)	C20—C21—C22	120.4 (3)
C19—N1—Mn1	124.21 (18)	C20—C21—H21A	119.8
C15—N1—Mn1	119.35 (18)	C22—C21—H21A	119.8
C24—N2—C20	116.1 (2)	C23—C22—C21	116.0 (2)
C24—N2—Mn1^iii^	120.75 (18)	C23—C22—C17	123.0 (2)
C20—N2—Mn1^iii^	123.16 (18)	C21—C22—C17	121.0 (2)
C25—N3—C29	116.8 (3)	C24—C23—C22	119.9 (3)
C34—N4—C30	115.1 (3)	C24—C23—H23A	120.1
O1—C1—O2	120.0 (3)	C22—C23—H23A	120.1
O1—C1—C2	119.9 (3)	N2—C24—C23	124.2 (3)
O2—C1—C2	120.0 (3)	N2—C24—H24A	117.9
O1—C1—Mn1	59.82 (15)	C23—C24—H24A	117.9
O2—C1—Mn1	60.27 (15)	N3—C25—C26	123.4 (4)
C2—C1—Mn1	179.2 (2)	N3—C25—H25A	118.3
C3—C2—C7	118.4 (3)	C26—C25—H25A	118.3
C3—C2—C1	120.9 (3)	C25—C26—C27	120.0 (3)
C7—C2—C1	120.6 (3)	C25—C26—H26A	120.0
C2—C3—C4	120.9 (3)	C27—C26—H26A	120.0
C2—C3—H3A	119.6	C28—C27—C26	116.1 (3)
C4—C3—H3A	119.6	C28—C27—C32	122.3 (3)
C5—C4—C3	119.8 (3)	C26—C27—C32	121.7 (3)
C5—C4—H4A	120.1	C29—C28—C27	119.6 (3)
C3—C4—H4A	120.1	C29—C28—H28A	120.2
O3—C5—C4	122.5 (3)	C27—C28—H28A	120.2
O3—C5—C6	117.3 (3)	N3—C29—C28	124.1 (4)
C4—C5—C6	120.2 (3)	N3—C29—H29A	117.9
C5—C6—C7	119.5 (3)	C28—C29—H29A	117.9
C5—C6—H6A	120.3	N4—C30—C31	124.5 (4)
C7—C6—H6A	120.3	N4—C30—H30A	117.7
C6—C7—C2	121.1 (3)	C31—C30—H30A	117.7
C6—C7—H7A	119.4	C32—C31—C30	119.7 (3)
C2—C7—H7A	119.4	C32—C31—H31A	120.1
O4—C8—O5	123.0 (3)	C30—C31—H31A	120.1
O4—C8—C9	119.3 (3)	C31—C32—C33	116.5 (3)
O5—C8—C9	117.7 (3)	C31—C32—C27	120.6 (3)
C10—C9—C14	118.6 (3)	C33—C32—C27	122.9 (3)
C10—C9—C8	121.2 (3)	C34—C33—C32	119.0 (3)
C14—C9—C8	120.2 (3)	C34—C33—H33A	120.5
C9—C10—C11	120.8 (3)	C32—C33—H33A	120.5
C9—C10—H10A	119.6	N4—C34—C33	125.1 (3)
C11—C10—H10A	119.6	N4—C34—H34A	117.4
C12—C11—C10	118.2 (3)	C33—C34—H34A	117.4

Symmetry codes: (i) −*x*, *y*, −*z*+3/2; (ii) *x*, *y*+1, *z*; (iii) *x*, *y*−1, *z*.

### Hydrogen-bond geometry (Å, °)

**Table:**

*D*—H···*A*	*D*—H	H···*A*	*D*···*A*	*D*—H···*A*
O1W—H1WA···O2W	0.94	2.00	2.781 (4)	140
O1W—H1WB···O2W^iv^	0.97	2.15	2.992 (4)	145
O2W—H2WB···N4	0.89	2.34	2.934 (4)	124
O2W—H2WA···O2	0.98	1.82	2.793 (3)	172
O3—H3B···N3^v^	0.82	1.88	2.694 (4)	169
O6—H6B···O1W^vi^	0.82	1.90	2.701 (4)	167

Symmetry codes: (iv) −*x*, −*y*, −*z*+1; (v) *x*+1, *y*, *z*; (vi) −*x*+1/2, *y*+1/2, −*z*+3/2.
